# Unsolicited Pics and Sexual Scripts: Gender and Relationship Context of Compliant and Non-consensual Technology-Mediated Sexual Interactions

**DOI:** 10.3389/fpsyg.2021.673202

**Published:** 2021-07-19

**Authors:** Erin Leigh Courtice, Konrad Czechowski, Pari-Gole Noorishad, Krystelle Shaughnessy

**Affiliations:** Faculty of Social Sciences, School of Psychology, University of Ottawa, Ottawa, ON, Canada

**Keywords:** technology-mediated sexual interaction, sexting, sexual compliance, sexual consent, gender differences, relationships, traditional sexual script, token resistance

## Abstract

Technology-mediated sexual interaction (TMSI) refers to any partnered interaction that involves sending or receiving self-created, sexually explicit content using communication technology (e. g., sexting, cybersex). Most research on TMSI assumes that experiences are desired and consensual. However, it is likely that some people do not desire all their TMSI experiences but consent to them anyways (*compliance*), or experience *non-consensual* TMSIs. People also engage in TMSIs with different types of partners. According to the traditional sexual script (TSS), other-gender attracted women and men's non-consensual TMSI experiences should differ overall and depending on the relationship context of the experience. The goal of this study was to examine the role of sexual scripts in other-gender attracted women and men's non-consensual and compliant TMSI experiences with committed romantic partners (CRPs), known non-partners (KNPs), and strangers (Ss). Women (*n* = 331) and men (*n* = 120) completed an online survey with questions about lifetime prevalence of experiencing seven types of compliant and non-consensual TMSIs in each relationship context. Results of mixed ANOVAs revealed significant interactions: overall, more participants reported compliant TMSI with CRPs. More women than men had received a non-consensual TMSI from someone they were not in a committed relationship with, and more men than women reported sending non-consensual TMSIs to a stranger. Tests of unpaired proportions suggested that the prevalence of sending and receiving non-consensual TMSIs was discordant in the KNP and S contexts: both women and men received more non-consensual TMSIs from KNPs and Ss than the other-gender reported sending. Our findings suggest that gendered sexual scripts are evident in some, but not all, aspect of other-gender attracted women and men's compliant and non-consensual TMSI experiences.

## Introduction

Over the past two decades, people's use of digital technology for interpersonal sexual communication has become common (Benotsch et al., 2013). We use the term *technology-mediated sexual interaction* (TMSI) to refer to any interaction with a specified other person(s), that includes sending and/or receiving self-created, sexually explicit content using communication technology (Courtice and Shaughnessy, 2017). Technology-mediated sexual interaction is a behavioral domain that integrates popular constructs such as sexting, cybersex, and phone sex. Researchers have found a wide prevalence of TMSI in research with young adults (ranging from <25% to over 80% of all participants; e.g., Ferguson, 2011; Delevi and Weisskirch, 2013). This wide range is likely due in part to limitations in evaluating the context of TMSI. Specifically, people's TMSI activities can be: (i) desired and consensual (*consensual*); (ii) undesired and consensual (i.e., *compliant*); and/or (iii) undesired and non-consensual (i.e., non-consensual; Döring, 2014; Drouin and Tobin, 2014; Morelli et al., 2016; Krieger, 2017). People also engage in TMSI with committed romantic partners, known non-partners (someone who is known, but not a committed partner; e.g., casual sex partner), and strangers—but may do so at different prevalence rates (Shaughnessy and Byers, 2014). Sexual script theory suggests that women and men's experiences with non-consensual TMSIs may differ, such that women receive, and men send, more non-consensual TMSIs overall (Shaughnessy and Byers, 2014; Courtice and Shaughnessy, 2018). Information about the consent, partner, and gender context of TMSI will clarify the prevalence of TMSI overall and improve knowledge on the circumstances in which TMSI may lead to different outcomes. Thus, the purpose of this study was to examine cisgender, other-gender attracted^1^ women and men's non-consensual and compliant TMSI experiences with committed romantic partners, known non-partners, and strangers.

### Prevalence of TMSI

It appears likely that TMSI is a common experience among young people and adults. Researchers have examined the prevalence of various forms of TMSI—particularly, cybersex and sexting. Overall, researchers have found conflicting information about the proportion of people who have engaged in some form of TMSI. For instance, in their 2017 systematic review of TMSI research, Courtice and Shaughnessy found that the reported prevalence estimates of TMSI among young adults range from 20.5% in a sample of American 16–25 year-olds (Ferguson, 2011) to 89.1% in a sample of American 18–30 year-olds (Delevi and Weisskirch, 2013). In their recent meta-analysis, Mori et al. (2020) found that the mean prevalence for sexting among emerging adults was 38.3% for sending, 41.5% for receiving, and 47.7% for exchanging sexts. Among adolescents, reported TMSI prevalence estimates range from 4.6% in a sample of American 10–15 year-olds (Rice et al., 2014) to 57.1% in a sample of Belgian 15–18 year-olds (Van Ouytsel et al., 2014). In their 2018 meta-analysis, Madigan and colleagues found that the mean prevalence for sexting among adolescents was 14.8% for sending and 27.4% for receiving sexts. These aforementioned ranges and mean prevalence rates do not take into account the consent or relationship context of TMSI. Indeed, it is not currently clear what proportion of people's TMSI experiences are fully desired and consensual.

### Consent Context of TMSI

#### Compliant TMSI

People can have TMSI experiences that are *compliant*: consented to, but not desired, by at least one person involved in the interaction. In our review of the research, we found only one study in which researchers distinguished between TMSIs that are enthusiastically consented to vs. compliant. Drouin and Tobin (2014) found that about half (52.3%) of their cisgender men and cisgender women in committed relationships had ever engaged in compliant sexting with current or previous partners. This finding is consistent with offline research that suggests that sexual compliance may be slightly higher for adults in committed romantic relationships compared to prevalence estimates that do not consider relationship context (e.g., Vannier and O'Sullivan, 2010). For instance, among a sample of sexually active college students, prevalence estimates of sexual compliance range from 6 to 38% (Vannier and O'Sullivan, 2010; Viscione, 2015); in Vannier and O'Sullivan's (2010) a daily diary study, 46% of heterosexual people in committed relationships reported sexual compliance at least once over a 2-week period. To our knowledge, no researchers have examined the prevalence of compliant TMSIs outside of the committed relationship context. Thus, it is unclear how common or uncommon compliant TMSI experiences are.

#### Non-consensual TMSI

There are several ways that TMSIs can be *non-consensual*. First, like offline sexual activity, people can be the victim (or recipient) of sexual activity for which they did not explicitly consent. Conversely, people can also be the perpetrator (or sender) of sexual activity that was not consented to. In other words, just like someone can sexually assault (and be sexually assaulted), someone can also send (and receive) non-consensual TMSIs. Second, the “technology-mediated” nature of TMSI yields new forms of non-consensual activities. Recently, Mori et al. (2020) defined non-consensual sexting as “forwarding of sexts without consent and those who were victims of the forwarding of sexts without consent.” This definition is narrow; there are other forms of non-consensual TMSI, including when someone shares a TMSI that was sent to them, without the sender's permission (non-consensual sharing) and when someone posts a TMSI that was sent to them online, without the sender's permission (“revenge porn” or non-consensual posting). It is possible that some people actually share or post TMSIs that they have received non-consensually as a way to respond to, perhaps cope with, this experience. Essentially, when someone receives a non-consensual TMSI, they may cope with that experience by involving others, sharing it to seek support, or posting it to shame the person who sent it (e.g., Waling and Pym, 2019; Naezer and van Oosterhout, 2020). Although all of these activities are distinct, they all have something in common: one person has not provided consent for the activity to occur.

Few researchers have examined the consent context of TMSI within studies on sexting or cybersex. In recent literature reviews, Krieger (2017) noted that only 28.1% of articles explicitly included non-consensual acts in researcher's operational definitions of sexting; Walker and Sleath (2017) identified only 18 empirical papers that examined the prevalence of non-consensual sharing of TMSIs. More recent research findings suggest that around 50% of adults reported receiving an unsolicited sexual message/image or genital image (Valiukas et al., 2019; Marcotte et al., 2020) and slightly fewer reported sending one (Oswald et al., 2019). In their literature review, Walker and Sleath (2017) found that between 1.1 and 6.3% of adult participants reported being the victim of non-consensual sharing (having a TMSI that one sent shared by someone else without the sender's consent), and between 1.4 and 16.3% reported sharing a TMSI they received from someone else without the sender's consent. There was only one study of non-consensual posting among adults; Hudson et al. (2014) found that 63.7% of participants reported ever having a TMSI posted online without their consent. Notably, these examinations have not looked at gender differences in people's experiences with non-consensual sharing and posting of TMSIs. Men are more likely than women to perpetrate sexual assault in person (Black et al., 2011)—thus, it is possible that men might also be more likely to share or post [women's] TMSIs without consent. On the other hand, some researchers have suggested that women may share or post TMSIs that they non-consensually received from men as a means of deterring men from perpetuating a non-consensual TMSI again in the future (Waling and Pym, 2019; Naezer and van Oosterhout, 2020). Thus, the gender context of non-consensually sharing and posting TMSIs to the internet is currently unclear. To address this gap, we explored the prevalence of women and men's experiences with sharing and posting TMSIs without the sender's consent, and having had their own TMSI shared and posted without consent (Research Questions 1 and 2).

There are a number of methodological limitations in the existing non-consensual TMSI research. First, as with the majority of research on TMSIs (e.g., Courtice and Shaughnessy, 2017), researchers have not used consistent terminology or methodology to measure non-consensual TMSI experiences. For instance, some researchers have limited their definitions of non-consensual sharing to images only (e.g., Bloom, 2014; Matsui, 2015), while others include both images and videos (e.g., Cecil, 2014; Cannon, 2015; Walker and Sleath, 2017). Indeed, restricting operational definitions of TMSI to one specific medium can lead to an inaccurate understanding of the extent to which people have experienced or engaged in the *behavior* of TMSI as a whole. To collect the most accurate information possible, it is important for researchers to include multiple mediums in their operational/conceptual definitions of the behavior itself. Additionally, there is some confusion about what behaviors actually constitute non-consensual TMSI. Some researchers include sending or receiving unsolicited sexual content in their definitions (e.g., Krieger, 2017); others limit their definitions to non-consensual sharing or posting of TMSIs (e.g., Morelli et al., 2016; Mori et al., 2020). Inconsistent definitions of non-consensual TMSI likely contribute to confusion about the overall prevalence of TMSI experience, and create difficulties for researchers who wish to make comparisons across studies. Second, no researchers (to our knowledge) have examined all forms of non-consensual TMSI within a single sample. As such, it is difficult to know how prevalent different forms of non-consensual TMSI are, relative to one another. Third, researchers have not compared the prevalence of receiving to sending of non-consensual TMSIs within a single population. It is possible that there are discrepancies in whether or not people experience TMSI as non-consensual depending on whether they are the perpetrator or receiver of the message. That is, someone may believe that a TMSI is consensual when they send it, even if it is not experienced as consensual by the person who receives the TMSI. We cannot understand the multiplicity of experiences if we focus only on one side of the interaction.

### Gender Differences in Mixed-Gender TMSI Experiences

#### Sexual Script Theory

Women and men likely differ in their non-consensual and compliant TMSI experiences. Sexual script theory is one approach for explaining how people learn about, internalize, and enact sexual behaviors—particularly social sexual behaviors (Wiederman, 2015). According to sexual script theory, people learn cultural-level scripts that act as guidelines for typical behaviors within a particular social-cultural context for sexual behaviors (Simon and Gagnon, 1984, 2003; Wiederman, 2015). These learned scripts tend to guide people's interpersonal sexual behavior, as well as inform expectations for their sexual experiences (Abelson, 1981; Simon and Gagnon, 1984, 2003; Simon, 1996).

In North America, the Traditional Sexual Script (TSS) is the dominant script guiding heterosexual, cisgender women, and cisgender men's sexual interactions (Crawford and Popp, 2003; Bartoli and Clark, 2006; Petersen and Hyde, 2010; Eaton and Rose, 2011; Sakaluk et al., 2014). Relevant studies published after 2015 suggest that university students do still endorse the TSS (Hust et al., 2017; Quinn-Nilas and Kennett, 2018; Rhodes, 2020; Marshall et al., 2021). Some researchers have suggested that the TSS also guides sexual minority people's sexual interactions (see Courtice and Shaughnessy, 2017; Gauvin and Pukall, 2018). The TSS emphasizes traditional gender roles for men and women in their sexual interactions (Wiederman, 2005; Masters et al., 2013). In doing so, the TSS places greatest acceptance on: (i) sexual behaviors within romantic relationships, (ii) men taking active roles and women passive roles, and (iii) using largely non-verbal communications of consent (Crawford and Popp, 2003; Bartoli and Clark, 2006).

#### Partner Context and the Traditional Sexual Script

According to the TSS, it is most acceptable for people to engage in sexual activity with a committed, and (often) monogamous partner (Byers, 1996; Wiederman, 2015; Gagnon and Simon, 2017). People do engage in consensual sexual activity outside of committed relationships; according to the TSS, these experiences can be acceptable as long as those involved are not in a committed relationship with someone else (Byers, 1996; Wiederman, 2015; Gagnon and Simon, 2017). Researchers have suggested that heterosexual people expect to and do engage in consensual sexual activity with people they know better (e.g., a committed partner or “friend with benefits”) compared to people they know less well (e.g., a casual acquaintance or stranger; Waite and Joyner, 2001; Birnie-Porter and Hunt, 2015). Furthermore, some researchers have suggested that heterosexual people's (presumably) consensual TMSIs follow this same pattern; people report the highest prevalence of TMSI with a committed romantic partner, followed by a known non-partner, and then a stranger (Shaughnessy and Byers, 2014). Because women are most likely to comply with and initiate sexual activity within the relationship context that is most acceptable for them, women should report the highest instances of TMSI in the committed romantic partner context. On the other hand, the TSS dictates that men should comply with and initiate sexual activity in all partner contexts (Byers, 1996; Wiederman, 2015). Because the committed romantic partner context is the most accessible for men to engage in sexual activity (and the context in which women are most likely to initiate), men are most likely to engage in TMSI in this context—even if the TMSI is undesired. Therefore, we hypothesized that more people would report compliant (consensual but unwanted) TMSI exchanges with a committed romantic partner, followed by a known non-partner, and then a stranger (Hypothesis 1).

#### Gender and the Traditional Sexual Script

A central tenet of the TSS is that men and women learn and enact different sexual scripts that place them in complementary yet opposing roles (Simon and Gagnon, 1984; Willetts et al., 2004; Sakaluk et al., 2014). Some researchers have highlighted how these scripted roles likely contribute to “token resistance” on the part of women, and persistence on the part of men (e.g., Muehlenhard and Lisa, 1988). Specifically, the script guiding women's sexual behaviors dictates that they should, at least initially, resist sexual activity even when they do wish to engage in it. This translates to a woman's initial sexual refusals being taken as merely tokenistic, and part of the script—a “no” can become a “yes” with additional persistence. Some men, then, respond to a woman's “no” with subsequent persistence, aiming to receive a “yes”—this male persistence, in turn, becomes part of the sexual script. The flip side of this token resistance script may be difficulty for women to continue saying “no” when they have already refused, and difficulty for men to hear refusals as final (Frith and Kitzinger, 2001). This difficulty may be particularly true within committed romantic relationships because of the cultural level script's acceptance of sexual behavior in these relationship contexts. Therefore, we hypothesized that more women than men would report lifetime prevalence of exchanging compliant TMSIs in all partner contexts (Hypothesis 2).

The TSS also is more accepting of men's sexual expression compared to women's. At the cultural level, men are encouraged to take an active role and desire, engage in, and thus pursue sexual behaviors with a wide range of partners—including with committed partners, known non-partners (e.g., “friends with benefits”), and strangers (Byers, 1996; Wiederman, 2015). In contrast, women are discouraged from desiring and engaging in sexual activity outside of a committed relationship context, such as with a known non-partner or stranger (McHugh et al., 2012). Applied to TMSIs, men should also pursue a variety of partner contexts, whereas women should only pursue TMSI with a committed romantic partner. Indeed, researchers have found that more men than women engage in TMSI with a wider variety of partner contexts; men also report more TMSI experience outside of the committed relationship context compared to women (Shaughnessy and Byers, 2014; Courtice and Shaughnessy, 2017). These findings suggest that men are more likely than women to initiate a TMSI exchange in known non-partner and stranger contexts. They also suggest that women are equally as likely as men to initiate a TMSI exchange in the committed relationship context only. With all the above in mind, we hypothesized that women would report greater lifetime prevalence of receiving (Hypothesis 3), and men would report a greater lifetime prevalence of sending (Hypothesis 4), non-consensual TMSIs in known non-partner and stranger contexts.

## The Present Study

The goal of this study was to examine the partner context of other-gender attracted women and men's compliant and non-consensual TMSI experiences, as an indicator of interpersonal sexual scripts. To address these aims, we examined the prevalence of non-consensual and compliant TMSIs as indicators of interpersonal sexual scripts. Specifically, we examined multiple forms of TMSIs: non-consensual sending, receiving, sharing, and posting, as well as compliant exchanges (i.e., back-and-forth sending and receiving). We also examined the prevalence of men and women's experiences in three relationship contexts: committed romantic partners, known non-partners (someone who is known, but not a committed partner; e.g., casual sex partner), and strangers. Based on sexual script theory, token resistance theory, and past research on TMSIs and offline sexual coercion, we examined the following four hypotheses:

1. Participants will report greater lifetime prevalence of exchanging compliant TMSIs with a committed romantic partner, then a known non-partner, followed by a stranger.
2. Relative to men, women will report greater lifetime prevalence of exchanging compliant TMSIs in all partner contexts.
3. Relative to men, women will report greater lifetime prevalence of receiving non-consensual TMSIs in the known non-partner and stranger contexts
4. Relative to women, men will report greater lifetime prevalence of sending non-consensual TMSIs in the known non-partner and stranger contexts.

Because of the lack of research with adults on non-consensual sharing and posting, we explored the following two research questions:

1. Do women and men differ in their experiences of having ever had someone share their TMSIs with another person or post their TMSI publicly across the three relationship contexts?
2. Do women and men differ in their experiences of non-consensually sharing or posting another person's TMSI across the three relationship contexts?

We also explored potential discrepancies between women and men's reported prevalence of sending, receiving, sharing, and posting non-consensual TMSIs. Our goal here was to examine the concordance/discordance of reporting for each activity (and in each partner context), to explore the possibility that women and men's perceptions of consensual/non-consensual TMSIs might differ. Specifically, we posed the following additional research questions:

3. In each partner context, will women and men report prevalence rates of receiving non-consensual TMSIs that are similar to those for sending non-consensual TMSIs?
4. In each partner context, will women and men report prevalence rates of having had a TMSI non-consensually shared and/or posted that are similar to those for non-consensually sharing and/or posting someone's TMSI?

## Methods

### Participants

A total of 671 people completed this online survey study. We identified 56 participants as duplicate responders and only their first response was retained for analyses. We excluded an additional 71 participants from analyses because they either completed the survey in under 5 min (*n* = 47) or in over 24 h (*n* = 24; Huang et al., 2012; Meade and Craig, 2012). We excluded 20 participants because they responded “no” to a question asking if their survey answers were honest. Because of the hetero- and cis-centric nature of our hypotheses, we included only cisgender women and men (4 participants excluded), and people with mostly/entirely other-gender sexual attraction (97 participants excluded).

Of the 451 participants retained for analyses, 331 (73.4%) identified as cisgender women and 120 (26.6%) as cisgender men. Participants ranged in age from 16 to over 30 years old (*M* = 19.7, *SD* = 3.0). Most participants identified as heterosexual (95.8%) and entirely sexually attracted to another (binary) gender (76.5%). Most were not in a committed relationship (55.7%). Additional sample demographic and background characteristics are reported in Table 1.

**Table 1:** Demographic and background characteristics of sample retained for analyses.

	**Subsample size (*n*)**	**Proportion of total sample (%)**
**Gender**		
Man	120	26.6
Woman	331	73.4
**Sexual Attraction**		
Entirely to other gender	345	76.5
Mostly to other gender	106	23.5
**Sexual Orientation**		
Heterosexual	432	95.8
Bisexual	6	1.3
Other	13	2.9
**Relationship Status**		
Committed relationship	191	42.4
Single and dating	58	12.9
Single and not dating	196	43.5
Prefer not to answer	6	1.3
**Country of Birth**		
Canada	362	80.3
China	17	3.8
Haiti	5	1.1
Other (e.g., France, Mexico, Egypt)	62	13.7
Prefer not to answer	5	1.1
**Age**		
16–17	17	3.0
18	209	36.8
19	143	25.2
20	74	13
21	52	9.2
22–24	38	6.7
25–29	18	3.3
30+	11	1.9

*Participants who endorsed the maximum “30+” option for age and were coded as 30 years old for mean and standard deviation calculations*.

### Procedure

Participants were recruited from a large University in Ontario, Canada to take part in an online survey about their “perspectives on sexual behaviors, dating habits, and dating scripts” from September 2017 to April 2018. All participants were university students enrolled in an introductory psychology course, who voluntarily registered for this study (from a list of open research studies) in exchange for course credit. Upon registering, participants received a link to the study survey, hosted on Qualtrics.

The first page of the survey was an Informed Consent Form, which provided information about the study, participants' rights, privacy, confidentiality, and information about data management and storage. Participants actively clicked to indicate their consent and were then directed to the online survey. Next, consenting participants completed background/demographic questions, which included questions about participant's gender and other sexual experiences that were not related to this study's objectives. Then, participants were asked about their experiences with non-consensual and compliant TMSIs in different partner contexts. On the final page of the survey, we provided participants with debriefing information. This study was approved by our institution's Research Ethics Board.

### Measures

#### Background Questionnaire

Participants responded to closed-ended questions based on previous research that assessed demographic and background information, including participants' gender, sexual identity, gendered sexual attraction, relationship status, sexual experience, and online dating app use. The questions about gender, sexual identity, and relationship status all had open-ended response options if the options available did not appropriately address one's identity or status. Participants were also able to skip any questions that they did not wish to answer.

#### Compliant and Non-consensual TMSI Experience

We created a 14-item measure to evaluate whether or not participants had ever engaged in a compliant TMSI exchange and/or sent, received, shared, or posted TMSIs that were non-consensual with each of three types of partners (i.e., committed romantic partner, known non-partner, and unknown other; see Table 2). We assessed each subtype of non-consensual/compliant TMSI experience using a checklist response format presented as a matrix table; participants selected each of the activities that they had experienced in their lifetime, as well as the type(s) of partner(s) they had experienced each activity with. We provided the definition for each type of partner in the instructions based on those used in previous research (Shaughnessy and Byers, 2014). For each activity, we used two items: one item specified *text* messages and the other item specified *photo/video* messages within this matrix to create dichotomous scores (0 = no, 1 = yes) for prevalence of compliant and non-consensual experience of TMSIs in each partner context. We combined responses to the text and photo/video items to create one dichotomous prevalence score (0 = no to both text and photo/video experiences; 1 = yes to either/both a text or photo/video experience; also reported as mean scores in Table 3) for each partner type, based on their conceptual face validity. Our focus was on assessing the compliant context rather than the specific medium of the experience. Thus, this summary prevalence score from multiple items ensured we asked about more than one mode, and allowed us to focus on whether the experience was compliant, or non-consensual (or not). Thus, we examined seven subtypes of non-consensual/compliant TMSI experience: (1) compliant TMSI (e.g., exchanged sexually explicit messages with another person when you did not want to), (2) non-consensual sending (e.g., sent a sexually explicit message to someone when they did not ask for it), (3) non-consensual receiving (e.g., received a sexually explicit message from someone when you did not ask them for it), (4) non-consensual sharing perpetration (e.g., showed a sexually explicit message that you received from someone else to another person, without the sender's permission), (5) non-consensual sharing victimization (e.g., had a sexually explicit message that you sent to someone else shared with another person without your permission), (6) non-consensual online posting perpetration (e.g., posted a sexually explicit message that you received from someone else to the internet, without the sender's permission), and (7) non-consensual posting victimization (e.g., had a sexually explicit message that you sent to someone else posted to the internet without your permission). We have presented the original items and their corresponding subtype in Table 2.

**Table 2:** Items comprising our measure of compliant and non-consensual TMSI experiences and their corresponding TMSI subtype.

**Original item**	**Compliant/non-consensual TMSI subtype**
1. Exchanged sexually explicit text messages via mobile phone or computer with another person when you did not want to	Compliant TMSI (1)
2. Exchanged sexually explicit photo or video messages via mobile phone or computer with another person when you did not want to	Compliant TMSI (1)
3. Sent a sexually explicit text message via mobile phone or computer when the person you sent it to did not ask for it	Non-consensual sending (2)
4. Sent a sexually explicit photo or video message via mobile phone or computer when the person you sent it to did not ask for it	Non-consensual sending (2)
5. Received a sexually explicit text message via mobile phone or computer when you did not ask for it	Non-consensual receiving (3)
6. Received a sexually explicit photo or video message via mobile phone or computer when you did not ask for it	Non-consensual receiving (3)
7. Shown a sexually explicit text message that you received from someone else to another person, without the sender's permission	Non-consensual sharing perpetration (4)
8. Shown a sexually explicit photo or video message that you received from someone else to another person, without the sender's permission	Non-consensual sharing perpetration (4)
9. Had a sexually explicit text message that you sent to someone else shared with another person without your permission	Non-consensual sharing victimization (5)
10. Had a sexually explicit photo or video message that you sent to someone else shared with another person without your permission	Non-consensual sharing victimization (5)
11. Posted a sexually explicit text message that you received from someone else to the Internet, without the sender's permission	Non-consensual online posting perpetration (6)
12. Posted a sexually explicit photo or video message that you received from someone else to the Internet, without the sender's permission	Non-consensual online posting perpetration (6)
13. Had a sexually explicit text message that you sent to someone else posted to the Internet without your permission	Non-consensual online posting victimization (7)
14. Had a sexually explicit photo or video message that you sent to someone else posted to the Internet without your permission	Non-consensual online posting victimization (7)

*When responding to the original items, participants were able to select as many options as applied from the following list: (1) Yes, with a primary partner; (2) Yes, with a known non-partner; (3) Yes, with an unknown non-partner; (4) No, I have never experienced this; (5) Prefer not to answer*.

**Table 3:** Prevalence of compliant and non-consensual TMSI experiences by gender.

	**Overall (*N* = 451) (%)**	***M* (*SD*)**	**Women (*n* = 331) (%)**	***M* (*SD*)**	**Men (*n* = 120) (%)**	***M* (*SD*)**	***p***	***ηp*^2^**
**Compliant TMSI**								
With a committed romantic partner	16.4	0.16 (0.37)_a_	15.1	0.15 (0.36)_a_	20.0	0.20 (0.40)_a_	0.216	0.00
With a known non-partner	15.7	0.16 (0.37)_a_	17.8	0.18 (0.38)_a_	10.0	0.10 (0.30)_ab_	0.044	0.01
With a stranger	5.3	0.05 (0.23)_b_	5.7	0.06 (0.23)_b_	4.2	0.04 (0.20)_b_	0.512	0.00
**Received a Non-consensual TMSI**								
From a committed romantic partner	17.7	0.18 (0.38)_a_	15.7	0.16 (0.36)_a_	23.3	0.23 (0.43)_a_	0.061	0.01
From a known non-partner	31.9	0.32 (0.47)_b_	35.6	0.36 (0.48)_b_	21.7	0.22 (0.41)_a_	0.005	0.02
From a stranger	33.3	0.33 (0.47)_b_	40.2	0.40 (0.49)_b_	14.2	0.14 (0.35)_a_	<0.001	0.06
**Sent a Non-consensual TMSI**								
To a committed romantic partner	22.0	0.22 (0.41)_a_	23.9	0.24 (0.43)_a_	16.7	0.17 (0.37)_a_	0.103	0.01
To a known non-partner	7.1	0.07 (0.26)_b_	8.2	0.08 (0.27)_b_	4.2	0.04 (0.20)_b_	0.145	0.01
To a stranger	2.7	0.03 (0.16)_b_	1.5	0.02 (0.12)_c_	5.8	0.06 (0.24)_a_	0.012	0.01
**Non-consensually sharing a TMSI**								
From a committed romantic partner	7.3	0.07 (0.26)_a_	7.6	0.08 (0.27)_a_	6.7	0.07 (0.25)_a_	0.750	0.00
From a known non-partner	17.3	0.17 (0.38)_b_	18.1	0.18 (0.39)_b_	15.0	0.15 (0.36)_a_	0.439	0.00
From a stranger	9.1	0.09 (0.29)_a_	10.6	0.11 (0.31)_a_	5.0	0.05 (0.22)_a_	0.069	0.01
**Had a TMSI Non-consensually shared**								
By a committed romantic partner	5.5	0.06 (0.23)_a_	6.0	0.06 (0.24)_b_	4.2	0.04 (0.20)_a_	0.443	0.00
By a known non-partner	10.4	0.10 (0.31)_b_	10.3	0.10 (0.30)_b_	10.8	0.11 (0.31)_a_	0.863	0.00
By a stranger	3.5	0.04 (0.19)_a_	2.7	0.03 (0.16)_a_	5.8	0.06 (24)_a_	0.115	0.01
**Non-consensually posted a TMSI**								
From a committed romantic partner	0.9	n/a	0.6	n/a	1.7	n/a	n/a	n/a
From a known non-partner	0.9	n/a	0.6	n/a	1.7	n/a	n/a	n/a
From a stranger	0.9	n/a	1.2	n/a	2.5	n/a	n/a	n/a
**Had a TMSI Non-consensually posted**								
By a committed romantic partner	1.6	n/a	0.9	n/a	1.7	n/a	n/a	n/a
By a known non-partner	1.1	n/a	0.9	n/a	1.7	n/a	n/a	n/a
By a stranger	0.9	n/a	1.6	n/a	2.5	n/a	n/a	n/a

*Prevalence rates are reported by percentage. Means and their standard deviations are for lifetime prevalence scores on the measure of compliant and non-consensual TMSI experience. Response options are reported as 0 (never) and 1 (at least one time). Significant p-values (p ≤ 0.01) indicate significant gender differences, and different subscripts down a column within gender indicate a significant partner context difference (p ≤ 0.01). M(SD) scores represent mean scores for the dichotomous (0 = no, 1 = yes) prevalence for each category of compliant/non-consensual TMSI experience*.

### Analytic Approach

We hypothesized, broadly, that people's prevalence of experiencing compliant and non-consensual TMSI would differ based on participant gender and across partner contexts (Hypotheses 1–4). We used the same analytic approach to test each of these four hypotheses; we report means and standard deviations in Table 3. We were unable to examine our research questions about non-consensual posting in this way, because the proportion of participants with these experiences was too low (require >5 cases per cell; Kroonenberg and Verbeek, 2018); however, we were still able to report the overall prevalence of having a TMSI non-consensually posted by someone else, and non-consensually posting another person's TMSI. For the remaining five subtypes, we conducted a 2 (gender) ×3 (partner context) mixed analysis of variance (ANOVA) using the prevalence of the TMSI experience. For each ANOVA, we used the corresponding subtype item (from our seven items measuring compliant and non-consensual TMSI experience) as the repeated dependent variable (Lunney, 1970; Myers et al., 1982). Because Mauchley's test of sphericity was violated for all analyses, we used the Huynh-Feldt correction to interpret results. Lüpsen and Rechenzentrum (2019) also recommend using the Huynh-Feldt correction when conducting an ANOVA with a binary dependent variable with unequal cell counts. As per Overall (1980), it is appropriate to use a between-within ANOVA for dichotomous variables with unequal cell sizes, but that a more conservative alpha level should be used—therefore, we used a cutoff of alpha = 0.001 for our five omnibus tests. We followed up significant interactions in two ways. First, to examine whether women and men differed in their prevalence of the TMSI experience within each partner context, we conducted follow-up comparisons using a Bonferroni correction. Second, we tested simple main effects for the effect of partner context for both women and men. For significant simple effects we used follow-up multiple comparisons to locate the differences between partner context within gender. For all tests, we used 95% confidence intervals with a 5% error level and a Bonferroni correction. A *post-hoc* sensitivity analysis with an alpha = 0.01 and power = 0.80 indicated that the minimum detectable effect size (Cohen's *f* ) for our sample was 0.066.

We examined the concordance/discordance between participants' self-reported receiving and sending of non-consensual TMSIs (Research Question 3), and prevalence of having had TMSIs non-consensually shared and non-consensually sharing another person's TMSIs (Research Question 4). For each relationship context, we conducted 2 (gender) ×2 (RQ3: sending/receiving, RQ4: had shared/sharing) unpaired proportion comparisons (Campbell, 2007; Richarson, 2011). We defined concordance as one gender (e.g., women) reporting a statistically similar (indicated by %_*diff*_ absolute value scores close to 0) prevalence of receiving non-consensual TMSIs relative to the other gender (e.g., men) reported prevalence of sending in a given relationship context. We defined discordance as one gender (e.g., women) reporting a statistically significant difference (indicated by %_*diff*_ absolute value scores above 0) in prevalence of receiving non-consensual TMSIs relative to the other gender (e.g., men) reported prevalence of sending in a given relationship context.

## Results

### Preliminary Analyses

Initial cleaning and screening procedures revealed no missing data on key background items (gender, sexual orientation, and sexual attraction) or the items used to measure compliant and non-consensual TMSI prevalence. We did not identify any univariate or multivariate outliers on the measure of compliant and non-consensual TMSI experience.

Prior to testing our hypotheses, we examined the percentage of participants who indicated they had a compliant or non-consensual TMSI experience. The majority of participants (68.5%) reported at least one experience with complaint or non-consensual TMSIs. Of these, 49.9% reported more than one experience with compliant or non-consensual TMSIs. Overall, the prevalence of compliant and non-consensual TMSI experiences ranged from 0 to 14 experiences (out of a possible 21) across all relationship contexts (*M* = 2.12, *SD* = 2.30). The percentage of women and men who had each type of experience for each partner context is reported in Table 3. Overall, few had engaged in compliant TMSI with a committed romantic partner (16.4%), known non-partner (15.7%) or stranger (5.3%). Some participants had received a non-consensual TMSI from a committed romantic partner (16.7%), known non-partner (31.9%) or stranger (33.3%), and some had sent a non-consensual TMSI to a committed romantic partner (22.0%), known non-partner (7.1%) or stranger (2.7%).

### Prevalence of Exchanging Compliant TMSIs

We tested whether more participants reported engaging in compliant TMSI with a committed romantic partner compared to a known non-partner or stranger (Hypothesis 1), and whether more women reported a compliant TMSI experience, in all partner contexts, compared to men (Hypothesis 2). The main effect for gender was not significant, *F*_(1,449)_ = 0.41, *p* = 0.524, η_*p*_^2^ = 0.001; the main effect for partner context was significant, *F*_(1.95,874.56)_ = 17.10, *p* < 0.001, η_*p*_^2^ = 0.037. The interaction between gender and partner context was not significant, *F*_(1.95,874.56)_ = 4.11, *p* = 0.018, η_*p*_^2^ = 0.009. Multiple comparisons revealed that significantly more people reported prevalence of engaging in a compliant TMSI exchange with a committed romantic partner compared to with a stranger (*M*_CRP-S_ = 0.126, *p* < 0.001, 95% CI [0.074, 0.178], η_*p*_^2^ = 0.081), and with a known non-partner compared to with a stranger (*M*_KNP-S_ = 0.090, *p* < 0.001, 95% CI [0.040, 0.139], η_*p*_^2^ = 0.081). There were no significant differences between the committed romantic partner and known non-partner contexts (see Table 3). Thus, our findings partially supported Hypothesis 1: significantly more men and women reported a compliant TMSI experience with a committed partner compared to a stranger, but not compared to a known non-partner. In contrast to Hypothesis 2, there were no significant gender differences in committed romantic partner or stranger contexts.

### Prevalence of Receiving Non-consensual TMSIs

We tested whether more women reported receiving non-consensual TMSIs compared to men in the known non-partner and stranger contexts (Hypothesis 3). The main effects of both gender and partner context were significant, *F*_(1,449)_ = 13.97, *p* < 0.001, η_*p*_^2^ = 0.030 and *F*_(1.98,888.46)_ = 4.85, *p* = 0.008, η_*p*_^2^ = 0.011, respectively. The interaction between partner context and gender was also significant, *F*_(1.98,888.46)_ = 14.65, *p* < 0.001, η_*p*_^2^ = 0.032.

Follow up analyses revealed that significantly more women than men reported receiving non-consensual TMSIs from known non-partners (*M*_*Women*−*Men*_= −0.14, *p* = 0.005, 95% CI [−0.24, −0.04], η_*p*_^2^ = 0.018) and from strangers (*M*_*Women*−*Men*_ = −0.26, *p* < 0.001, 95% CI [−0.356, −0.164], η_*p*_^2^ = 0.060); thus, the interaction qualified the main effect of gender. In line with Hypothesis 3, significantly more women than men reported receiving a non-consensual TMSI from a known non-partner and from a stranger. There were no significant gender differences in the committed romantic partner context.

Because scripts also vary along partner context, we conducted a *post-hoc* follow-up analysis to explore the interaction by way of the partner context main effect as well. The follow up revealed that the main effect of partner context was significant only for women [*F*_(2,448)_ = 35.326, *p* < 0.001, η_*p*_^2^ = 0.14] and not for men [*F*_(2,448)_ = 1.519, *p* = 0.220, η_*p*_^2^ = 0.007]. Significantly more women reported prevalence of receiving non-consensual TMSIs within the known non-partner context (*M*_KNP-CRP_ = 0.20, *p* < 0.001, 95% CI [0.13, 0.27], η_*p*_^2^ = 0.136) and within the stranger context (*M*_S-CRP_ = 0.26, *p* < 0.001, 95% CI [0.17, 0.32], η_*p*_^2^ = 0.136) relative to the committed romantic partner context. There were no significant differences for women between the known non-partner and the stranger context (see Table 3).

### Prevalence of Sending Non-consensual TMSIs

We tested whether more men reported sending non-consensual TMSIs compared to women in the known non-partner and stranger contexts (Hypothesis 4). The main effect for gender was not significant, *F*_(1,449)_ = 1.30, *p* = 0.254, η_*p*_^2^ = 0.003; the main effect for partner context was significant, *F*_(1.63,733.27)_ = 36.11, *p* < 0.001, η_*p*_^2^ = 0.074; the interaction between gender and partner context was not significant, *F*_(1.63,733.27)_ = 3.99, *p* = 0.027, η_*p*_^2^ = 0.010. Multiple comparisons revealed that significantly more people reported prevalence of sending a non-consensual TMSI to a committed partner compared to a known non-partner (*M*_CRP-KNP_ = 0.141, *p* < 0.001, 95% CI [0.084, 0.198], η_*p*_^2^ = 0.103) and a stranger (*M*_CRP-S_ = 0.166, *p* < 0.001, 95% CI [0.110, 0.222], η_*p*_^2^ = 0.103). There were no significant differences between the known non-partner and stranger contexts (see Table 3). Thus, we did not find support for Hypothesis 4; there were no differences in women and men's prevalence of sending non-consensual TMSIs.

### Prevalence of Non-consensual Sharing of TMSIs

We explored whether women and men differed in their experiences of having ever had TMSIs they sent to another person non-consensually shared by that person, across the three relationship contexts (Research Question 1). The main effect for gender was not significant, *F*_(1,449)_ = 0.130 *p* = 0.719, η_*p*_^2^ = 0.000; the main effect for partner context was significant, *F*_(1.94,869.90)_ = 7.648, *p* = 0.001, η_*p*_^2^ = 0.017;(1.92, 869.90) = 1.024, *p* = 0.358, η_*p*_^2^ = 0.002. There were no significant differences between the committed romantic partner and stranger contexts (see Table 3).

We also explored whether women and men differed in their experiences of having ever non-consensually shared TMSIs they had received from another person across the three relationship contexts (Research Question 2). The main effect for gender was not significant, *F*_(1,449)_ = 2.272, *p* = 0.132, η_*p*_^2^ = 0.005; the main effect for partner context was significant, *F*_(1.92,862.05)_ = 11.181, *p* < 0.001, η_*p*_^2^ = 0.024; the interaction between gender and partner context was not significant, *F*_(1.92,862.05)_ = 0.533, *p* = 0.569, η_*p*_^2^ = 0.001. There were no significant differences between the committed romantic partner and stranger contexts (see Table 3).

### Concordance/Discordance of Non-consensual Receiving, Sending, and Sharing TMSIs

A side-by-side comparison of prevalence for sending and receiving non-consensual TMSIs and summary statistics for each unpaired proportion calculation are presented in Table 4. The prevalence of sending and receiving non-consensual TMSIs was concordant in the committed romantic partner context: women received as many non-consensual TMSIs from a committed romantic partner as men reported sending to a committed romantic partner (*x*^2^ = *0.0*17, *p* = *0.8*95), and men received as many non-consensual TMSIs as women reported sending (*x*^2^ = *0.0*65, *p* = *0.7*98). The prevalence of sending and receiving non-consensual TMSIs was discordant in the known non-partner context: women received more non-consensual TMSIs from known non-partners than men reported sending to known non-partners (*x*^2^ = 43.71, *p* < *0.0*01), and men received more non-consensual TMSIs than women reported sending (*x*^2^ = 15.40, *p*< *0.0*01). The prevalence of sending and receiving non-consensual TMSIs also was discordant in the stranger context; women received more non-consensual TMSIs from strangers than men reported sending to strangers (*x*^2^ = 48.58, *p* < *0.0*01), and men received more non-consensual TMSIs than women reported sending (*x*^2^ = 30.54, *p* < *0.0*01).

**Table 4:** Prevalence of sending and receiving non-consensual TMSIs.

	**Men sending**	**Women receiving**	**Women sending**	**Men receiving**	**Overall sending**	**Overall receiving**
**With a committed romantic partner**						
Prevalence (%)	16.7	15.7	23.9	23.3	22.0	17.7
%*_*diff*_* (95% CI)	1.0 (−6.08; 9.45)		0.60 (−8.76; 8.86)		4.30 (−0.91; 9.49)	
*x*^2^	0.065		0.017		2.62	
*p*	0.798		0.895		0.106	
**With a known non-partner**						
Prevalence (%)	4.2	35.6	8.2	21.7	7.1	31.9
%*_*diff*_* (95% CI)	31.4 (24.19; 37.21)		13.50 (6.20; 22.06)		24.80 (19.84; 29.68)	
*x*^2^	43.71		15.40		88.26	
*p*	<0.0001		<0.0001		<0.0001	
**With a stranger**						
Prevalence (%)	5.8	40.2	1.5	14.2	2.7	33.3
%*_*diff*_* (95% CI)	34.4 (26.72; 40.53)		12.70 (7.20; 20.12)		30.60 (25.98; 35.22)	
*x*^2^	48.58		30.54		142.90	
*p*	<0.0001		<0.0001		<0.0001	

We examined the concordance/discordance between participants' self-reported non-consensual sharing and having TMSIs non-consensually shared (Research Question 4). A side-by-side comparison of prevalence for sharing and having shared non-consensual TMSIs and summary statistics for each unpaired proportion calculation are presented in Table 5. The prevalence of non-consensually sharing and having TMSIs non-consensually shared was concordant between women and men in all three partner contexts. That is, a similar percentage of women reported sharing someone else's TMSI as men reported having had their TMSI shared, and vice versa within committed romantic partner, known non-partner, and stranger contexts separately. However, a *post-hoc* comparison within the overall sample revealed that the prevalence of non-consensually sharing and having TMSIs non-consensually shared was discordant in both the known non-partner (*x*^2^ = *0.8*.99, *p* = *0.0*03) and stranger (*x*^2^ = 11.97, *p* < 0.001) contexts. In both partner contexts, more people reported non-consensually sharing TMSIs than people reported having had them shared.

**Table 5:** Prevalence of non-consensually sharing and having TMSIs non-consensually shared.

	**Men shared**	**Women had shared**	**Women shared**	**Men had shared**	**Overall sharing**	**Overall had shared**
**With a committed romantic partner**						
Prevalence (%)	6.7	6.0	7.6	4.2	7.3	5.5
%*_*diff*_* (95% CI)	0.7 (−3.80; 7.00)		3.4 (−2.35; 7.53)		1.8 (−1.44; 5.09)	
*x*^2^	0.074		1.63		1.22	
*p*	0.785		0.202		0.270	
**With a known non-partner**						
Prevalence (%)	15.0	10.3	18.1	10.8	17.3	10.4
%*_*diff*_* (95% CI)	4.7 (−1.79; 12.69)		7.3 (−0.49; 13.59)		6.9 (2.40; 11.41)	
*x*^2^	1.90		3.46		8.99	
*p*	0.168		0.063		0.003	
**With a stranger**						
Prevalence (%)	5.0	2.7	10.6	5.8	9.1	3.5
%*_*diff*_* (95% CI)	2.3 (−1.28; 7.93)		4.8 (−1.59; 9.61)		5.6 (2.45; 8.90)	
*x*^2^	1.45		2.40		11.97	
*p*	0.228		0.122		<0.0001	

## Discussion

The goal of this study was to examine the partner context of other-gender attracted women and men's compliant and non-consensual TMSI experiences, as an indicator of interpersonal sexual scripts. To our knowledge, this study is the first examination of multiple types of compliant and non-consensual TMSIs alongside the relationship context of these experiences. Our results revealed that people's experiences with compliant and non-consensual forms of TMSI vary as a function of gender, relationship context, and role within the exchange (e.g., as a sender vs. receiver). Specifically, we found that women and men's experiences were somewhat in line with the TSS, offline sexual consent research, and our hypotheses. However, we also identified important ways that our findings diverged from what is predicted by the TSS. These findings extend and improve upon the small body of research on compliant and non-consensual TMSIs.

### Prevalence of Compliant and Non-consensual TMSIs

Our findings substantially improve TMSI research by showing that compliant and non-consensual TMSIs occur across multiple relationship contexts and in many ways. Most researchers have not included the consent context of experiences in their measures of TMSI, even as a precaution to ensure that they are evaluating consensual TMSI (Krieger, 2017). Our findings suggest that researchers who have not distinguished the consent context of TMSIs in their studies have potentially reported inaccurate results—that is, researchers may have overestimated the extent to which people experience consensual TMSIs by also capturing compliant and non-consensual experiences. Indeed, we found that compliant and non-consensual TMSIs appear to be common experiences that researchers must account for. We found that the majority (68.5%) of our sample reported at least one experience with a compliant or non-consensual TMSI. Furthermore, research on compliant and non-consensual TMSI is almost always focused on only one or two behaviors (e.g., non-consensual sharing of TMSIs or receiving unsolicited sexual messages; Marcotte et al., 2020; Mori et al., 2020) or one relationship context (e.g., compliance in committed romantic relationships; Drouin and Tobin, 2014). In asking about many two-way (compliance) and one-way (non-consensual) experiences, we learned that many people have multiple experiences. Indeed, we found that about 57% of participants reported more than one compliant or non-consensual TMSI, and participants overall reported an average of three experiences across all relationship contexts. These statistics are similar to the prevalence range for analogous offline experiences (Vannier and O'Sullivan, 2010; Viscione, 2015). Thus, our results suggest that people's experiences with compliant and non-consensual TMSI likely are as prevalent as offline compliant and non-consensual sexual experiences—at least among young adults in Western, urban settings.

We also found that some types of non-consensual TMSI were not very prevalent: non-consensually sharing or posting someone else's TMSI and having a TMSI non-consensually shared or posted were uncommon experiences overall, and for both women and men. Thus, most people do not report this type of non-consensual TMSI despite some researchers focusing on the harms of TMSI as tied to non-consensual sharing (e.g., Mori et al., 2020). It is possible that participants struggled to answer our questions about being the victim of non-consensual TMSI sharing and posting because of the likely hidden nature of these experiences. In Canada—where our participants resided—it is illegal to both non-consensually share and post sexually explicit content that was intended to be a two-way exchange (Protecting Canadians from Online Crime Act, 2014). Therefore, it is unlikely that someone would directly inform another person that they had done either of these activities. Indeed, it is possible that some participants had been victims of these activities, but were not aware that they were victims. This is especially likely in the stranger context, where there may not have been further contact after the TMSI was sent/received initially. Similarly, despite the anonymous context of our survey, it remains possible that our participants would not admit to perpetrating these behaviors due to fear of possible legal repercussions. In future studies, researchers may need to use targeted sampling strategies or develop ways of disguising the questions to accurately evaluate and understand people's experiences.

### Sexual Script Theory and Sexual Compliance in TMSI

The results of our study provide some evidence that the TSS and token resistance theory characterize aspects of compliant TMSI experiences. Consistent with the relationship context of sexual experience in the TSS, we found that many of our participants reported compliant TMSI with someone that they knew—either a committed romantic partner or a known non-partner. This finding partially aligns with a focus on committed romantic relationships in compliant sexual activity research—both offline and in technology-mediated contexts (e.g., Vannier and O'Sullivan, 2010; Drouin and Tobin, 2014; Viscione, 2015). Our findings indicate that people do engage in compliant TMSI within and outside of committed romantic relationships. However, the findings were not consistent with our expectations for compliant TMSI based on the sexual script and token resistance theory. First, we did not find evidence of an overall difference between the prevalence of men and women's compliant TMSIs. This suggests that, in contrast to what token resistance scripts would predict, women and men may experience equal amounts of pressure to comply when a TMSI is initiated, especially when the partner is known to them (i.e., a known non-partner or committed romantic partner). Second, we found that about as many people reported engaging in compliant TMSI with a committed romantic partner as with a known non-partner—however, more people reported compliant TMSI in these contexts relative to the stranger context. This finding stands in contrast to previous offline research suggesting that sexual compliance may be slightly higher for adults in committed romantic relationships (e.g., Vannier and O'Sullivan, 2010; Viscione, 2015) —however, it is somewhat consistent with the TSS and token resistance scripts for people in general. That is, people—regardless of gender—may be just as likely to comply with partnered sexual activity when the other person is known to them—not just when they are in a committed relationship with that person. It is possible that a person may feel more pressure to engage in compliant sexual activity with a known non-partner relative to a stranger because of the added accountability that the known non-partner context creates. That is, it may be easier to brush off a stranger's solicitations, but more difficult to resist the solicitations of someone who is known. Overall, these findings suggest that aspects of women and men's sexual scripts related to sexual compliance may somewhat differ online. Conducting a closer examination of people's experiences with compliant TMSI in each of these relationship contexts might explain why compliant exchanges occur.

### Sexual Script Theory and Non-consensual TMSI

The results of our study provide evidence that the TSS can explain some—but not all—aspects of women and men's non-consensual TMSI experiences. Consistent with the TSS, we found that more women than men reported receiving non-consensual TMSIs from known non-partners and strangers. Similarly, we found that more men than women reported sending non-consensual TMSIs to strangers. These findings are consistent with researcher's focus on men sending and women receiving “unsolicited dick pics” (Marcotte et al., 2020), and the script that directs men to pursue sexual activity in multiple relationship contexts. We also found that women and men's reported prevalence of sending and receiving was discordant in the known non-partner and stranger contexts. That is, both men and women received more non-consensual TMSIs than they sent to known non-partners and strangers. There are several explanations for these findings.

First, it is possible that there is discordance between what the senders and receivers perceive to be a signal of consent. People who send non-consensual TMSIs to known non-partners and strangers may not believe that the TMSIs they sent were non-consensual, whereas the receivers do perceive those TMSIs as non-consensual. This presupposes that sending a non-consensual TMSI is an attempt to initiate sexual activity, and that senders may intend their messages to be subtle, non-verbal technology-mediated sexual initiations. Indeed, it is unclear how people navigate consent for or during TMSIs. In fact, no researchers (to our knowledge) have examined or theorized on how people navigate consent for sending and receiving TMSIs generally. Because traditional indicators of non-verbal consent (e.g., direct eye contact, pulling someone closer) or non-consent (e.g., shaking head “no,” pushing someone away) are less available in TMSI, people may be more likely to bypass the consent process entirely and instead rely on what they expect based on the TSS—such as by sending a sexually explicit TMSI without asking directly. However, it is possible that subtlety is lost when communicating via technology rather than in-person. People—especially men—do seem to use mostly non-verbal cues when communicating sexual consent, particularly outside of the committed romantic partner context and with strangers (Muehlenhard et al., 2016). We also found that there were no gender differences in sending or receiving non-consensual TMSIs from committed romantic partners. This suggests that people may have a better understanding of a committed partner's sexual consent signals, both in terms of knowing a partner's intent and when they would be violating each other's consent.

Second, it may have been difficult for participants to admit to perpetrating non-consensual TMSI against known non-partners or strangers when they were completing this study. However, because the TSS dictates that pursuing sexual activity in a committed romantic context is permissible for both men and women, people may have been more honest in reporting their non-consensual TMSIs in this context, perhaps not viewing non-consensual TMSIs as a transgression. Indeed, according to the TSS, it is unacceptable for women to initiate sexual activity outside of a committed romantic relationship; knowledge of this script may prompt women to respond dishonestly, by reporting that they have not pursued sexual activity in known non-partner and stranger contexts. However, this explanation does not hold for men. On the other hand, it is also possible that only a small proportion of women and men may be responsible for sending the majority of non-consensual TMSIs in known non-partner and stranger contexts.

Finally, it is plausible that people send non-consensual sexual material to assert dominance over another, or as a hostile act of violence (Oswald et al., 2019). Indeed, when women and men reported non-consensual sharing and victimization, it was more likely with a known non-partner than a committed romantic partner or a stranger. It is unlikely that a large proportion of people would non-consensually share an image sent to them by a committed partner, with whom they have established relationship norms and trust. Of course, non-consensual sharing can be perpetrated by a committed romantic partner, but this is not likely to be a common behavior for a current and ongoing relationship. However, it seems that there may be something about the known non-partner context that is related to a lower respect for another person's privacy relative to strangers. It is possible that people are more likely to be honest about having non-consensually shared a known non-partner's TMSIs compared to the other two contexts—for instance, because known non-partners (e.g., possibly a former committed partner) might have more desire to hurt or enact vengeance upon the victim relative to committed romantic partners and strangers. Our findings about non-consensual sharing may also be an artifact, stemming from the generally low prevalence of people who reported sending non-consensual TMSIs to strangers.

## Limitations and Future Directions

The present study is not without limitations. First, our sample consisted of young adults who were attending a large Canadian University. As a result, we don't know if these results generalize to older people who live outside of Canada and who have different levels of education. Future studies should examine the compliant and non-consensual TMSI experiences among more diverse (in age, gender, sexual identity, country of residence, education level, and sexual experience) groups of adults. Second, our methodology relied on a self-report measure of compliant and non-consensual TMSI experiences and was therefore susceptible to reporting bias. Indeed, we identified discordant responses between items measuring non-consensual sending and receiving and non-consensual sharing and having TMSIs non-consensually shared. It is unclear whether or not this discordance is a result of dishonest reporting by participants. However, the anonymous context for our survey likely decreased the potential for this particular bias. Third, the three relationship contexts we examined are limited in capturing with whom people might experience compliant or non-consensual TMSI. We did not account for the possibility that different relationship contexts could apply to the same person—for example, people might have engaged in compliant TMSI with someone who was a committed romantic partner at first, but who then became a known non-partner over time. It is possible that participants reported experience with both types of relationship contexts, even if the activity occurred with only one person. In future studies, researchers could examine compliant and non-consensual TMSI experiences throughout the course of people's relationships. We also only accounted for the gender of participants in our analyses—we did not collect information about the other person involved in the compliant or non-consensual TMSIs. Researchers examining the potential role that gender plays in people's TMSI experiences should aim to collect information about the gender of all people involved in a compliant or non-consensual TMSI exchange. Similarly, because our focus was solely on people's compliant and non-consensual TMSI experiences, we did not ask participants about their experiences with desired and consensual TMSIs. In the future, researchers should aim to collect information about people's experiences with TMSI in all consent contexts; this information could shed further light on who is most likely to report TMSI in each context and the relative prevalence of each experience. Fourth, although our questions were framed to address non-consensual sending and receiving from a sexual partner, it is possible that people interpreted our questions to be about sending and receiving non-consensual TMSIs from anyone. For example, someone might share a received sexual image with a friend, without consent from (a) the person depicted in the image, nor (b) the friend that the image was sent to. It is possible that a misinterpretation of our question could partly explain our results related to gender differences in non-consensual sending and receiving of TMSIs. However, researchers have previously found that there are no differences between men and women in non-consensual sharing of TMSIs (Garcia et al., 2016; Madigan et al., 2018; Molla-Esparza et al., 2020). We also did not find gender differences in the prevalence of non-consensual sharing of TMSIs. Finally, although our results can be explained by the TSS and sexual script theory, this is one theory of many that could be useful in understanding these results. Indeed, we used people's gender and experiences with compliant and non-consensual TMSIs in multiple partner contexts as an indicator of sexual script endorsement. In the future, researchers may wish to directly assess people's endorsement of the TSS alongside their experiences with compliant and non-consensual TMSIs.

## Conclusion

To our knowledge, this is the first study that includes multiple consent contexts of TMSI, as well as the different relationship contexts in which people have experienced them. The results of our study indicate that both participant gender and the relationship context of the interaction are important factors in understanding people's compliant and non-consensual TMSI experiences. As such, our findings extend and contextualize previous research by highlighting the importance of addressing consent and relationship context when examining TMSI. By ignoring the possibility of non-consensual and compliant TMSIs, researchers have neglected the full scope of people's experience. Indeed, it is possible that the prevalence of *consensual* TMSIs—including sexting and cybersex—could be lower than researchers have previously reported. In a world where sexual interactions are increasingly mediated through technology, researchers must include the offline relationship context in their examinations of people's TMSI experiences. It is only in acknowledging the offline, technology-mediated, consensual, and non-consensual contexts of people's experiences that researchers can understand the full scope of people's technology-mediated sexual behaviors.

## Data Availability Statement

The datasets presented in this article are not readily available because unfortunately, at the time of data collection we did not include open science protocol in our informed consent procedures. Because our participants did not consent to having their raw data shared with other researchers, we are not able to ethically provide open data. We have added our measure of compliant and non-consensual TMSI experiences in-text (see Table 2), and would also be willing to provide our study materials to other researchers if contacted. Requests to access the datasets should be directed to krystelle.shaughnessy@uottawa.ca.

## Ethics Statement

The studies involving human participants were reviewed and approved by University of Ottawa Research Ethics Board. The patients/participants provided their written informed consent to participate in this study.

## Author Contributions

EC conceptualized the study and research methods, collected the data, conducted data analyses, wrote and edited all drafts of the manuscript, and prepared the manuscript for submission. KC conceptualized the study and research methods, collected the data, and edited several drafts of the manuscript. P-GN cleaned the data and ran some data analyses. KS assisted with study conceptualization, data analyses, supervised the project, and edited all drafts of the manuscript. All authors contributed to the article and approved the submitted version.

## Conflict of Interest

The authors declare that the research was conducted in the absence of any commercial or financial relationships that could be construed as a potential conflict of interest.

## Footnotes

^1^We use the term “other-gender attracted” so as not to exclude/erase those who are attracted to people that do not share their gender identity, but who are not heterosexual—for example, people who identify as bisexual, pansexual, and/or queer.
